# Passage of the Channel-Forming Agent Nystatin Through Ergosterol-Containing Lipid Membranes

**DOI:** 10.1007/s00232-025-00354-3

**Published:** 2025-07-07

**Authors:** Megi Tinev, Luka Kristanc, Gregor Gomišček, Bojan Božič

**Affiliations:** 1 Faculty of Medicine, Institute of Biophysics, University of Ljubljana, Vrazov trg 2, 1000 Ljubljana, Slovenia; 2 Faculty of Health Sciences, University of Novo mesto, Na Loko 2, 8000 Novo mesto, Slovenia; 3 Faculty of Health Sciences, University of Ljubljana, Zdravstvena pot 5, 1000 Ljubljana, Slovenia

**Keywords:** Phospholipid vesicle, Ergosterol, Nystatin, Transmembrane passage

## Abstract

The passage of nystatin through the ergosterol-containing phospholipid bilayer was studied on monovesicular and multivesicular giant unilamellar vesicles (GUVs and MVVs). Phase-contrast optical microscopy was used to examine vesicles composed of a palmitoyl-oleoyl-phosphatidyl-choline (POPC) bilayer with either 15 or 45 mol% ergosterol. Three types of vesicles were analyzed: (i) GUVs, (ii) outer vesicles (outGUVs) of MVVs, and (iii) inner vesicles (inGUVs) of MVVs. The times of their ruptures were determined after their exposure to nystatin at concentrations of 250 and 500 $\mu$M. At both concentrations, the times that the inGUVs spent in the nystatin solution after the rupture of the corresponding outGUVs were significantly shorter than the rupture times of individual GUVs of the similar size. These differences in rupture times demonstrate that the ergosterol-containing POPC membrane is permeable to nystatin.

**Graphical abstract:**

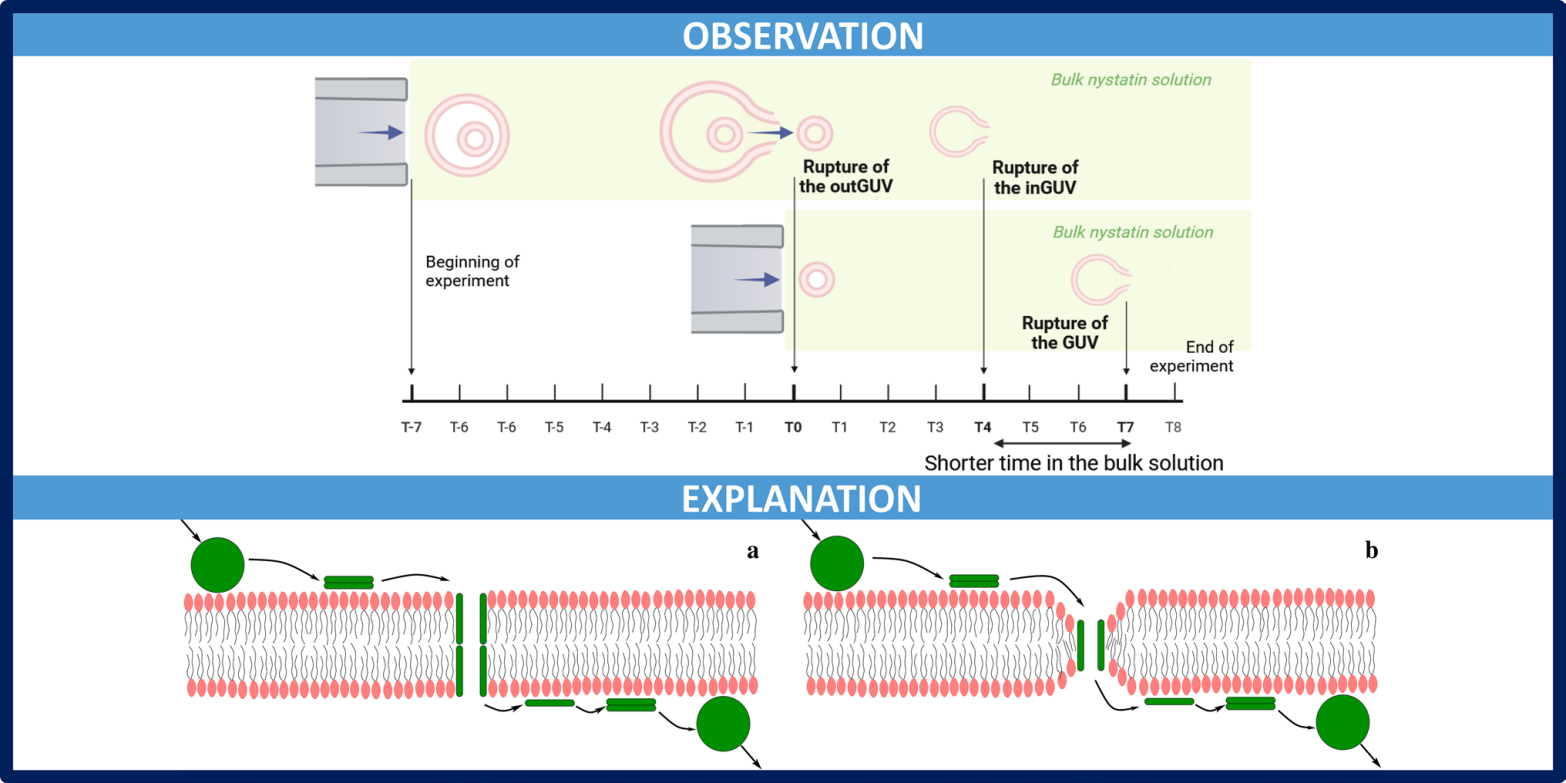

**Supplementary Information:**

The online version contains supplementary material available at 10.1007/s00232-025-00354-3.

## Introduction

Nystatin is a polyene antimicrobial agent with a broad spectrum of activity and relatively low resistance among fungal pathogens (Michel 1977; Bolard 1986; Ghannoum and Rice 1999). These properties make it one of the most important agents for the treatment of refractory fungal infections in humans (Ghannoum and Rice 1999). Beyond its antifungal applications, nystatin has demonstrated in vitro leishmanicidal activity against both promastigote and amastigote forms of several *Leishmania* species (Yamamoto et al. 2018), with successful in vivo results achieved through topical treatment in a BALB/c mouse model (Gonçalves-Oliveira et al. 2023). Thus, nystatin holds potential for topical applications against cutaneous infections caused by intracellular leishmanial pathogens. Structurally, nystatin exhibits an elongated shape with a length of approximately 2.8 nm and a large molecular weight (926 g/mol). Like other polyenes, it is chemically degradable at acidic pH (e.g., in the stomach) and poorly soluble in water and ethanol at 24 °C. Its solubility in methanol is slightly higher, but still limited due to formation of aggregates (Michel 1977; Akaike and Harata 1994).

Ergosterol intensifies the formation of nystatin transmembrane channels, which leads to an increase in the permeability of the cell membrane and ultimately to cell death (Hammond 1977; Bolard 1986). Since ergosterol is a major component of the fungal cell membrane (Weete et al. 2010) and of the membranes of certain kinetoplastid protozoans, such as the parasitic *Leishmania* and *Trypanosoma* (Roberts et al. 2003), the effect of nystatin in these cells is more intense than in the animal or plant cells, in which cholesterol is a key component. However, nystatin also has a certain affinity for cholesterol and, as a result, it can accumulate in certain organs, particularly the kidneys, leading to moderate or even severe toxicity (Hammond 1977; Zager 2000). Therefore, nystatin is primarily used topically to treat mucocutaneous candidiasis or administered parenterally in less nephrotoxic liposomal formulations to treat invasive fungal and leishmanial infections, especially in immunocompromised patients (Sousa et al. 2023). Currently, oral nystatin formulations for systemic use, such as nanosuspensions and cochleate formulations, are researched. These advances could enable patients to self-administer treatments for systemic fungal infections (Sousa et al. 2023). The prerequisites for an effect of nystatin should therefore be an efficient transition of its molecules from the intestines into the bloodstream, minimal toxicity to human cells and high activity in the membranes of pathogens. The latter include extracellular pathogens, such as the extracellular stages of pathogenic fungi and trypomastigote, as well as intracellular pathogens, such as *Leishmania* and *Trypanosoma* amastigotes and fungal pathogens in host cells.

Knowledge of the mechanisms by which nystatin molecules bind and form channels in the lipid membrane as well as pass through the lipid membrane is important. Numerous studies have researched the effects of nystatin on lipid membranes using various fluorescence and phase contrast microscopy techniques (Haro-Reyes et al. 2022). Research was focused on the effect of nystatin on the membranes of small (Coutinho and Prieto 2003), large (Vertut-Croquin et al. 1983; Coutinho et al. 2004; Silva et al. 2006) and giant (Kristanc et al. 2012, 2014) unilamellar phospholipid vesicles containing varying concentrations of ergosterol and cholesterol. These studies primarily examined the binding of nystatin to the membranes and the consequences of its aggregation within them. Nystatin was found to be more soluble in membranes containing ergosterol than in those containing cholesterol (Silva et al. 2006; Szomek et al. 2021). In addition, the extent of nystatin insertion into the lipid bilayer showed a complex, non-linear relationship with the increase in the sterol content of the membrane (González-Damián and Ortega-Blake 2010). Studies also showed that at sufficiently high concentrations, nystatin forms size-selective channels in the lipid membrane. Their size depends on the number of nystatin molecules present in the structure and, consequently, on the overall concentration of nystatin in the membrane (Haro-Reyes et al. 2022). These channels, consisting of 4–12 nystatin molecules, are structurally similar in the ergosterol- and cholesterol-containing membranes, with no significant differences observed in their radii (Kleinberg and Finkelstein 1984; Akaike and Harata 1994; Coutinho and Prieto 2003; Katsu et al. 2008). The nystatin-treated membranes are mainly permeable to molecules no larger than glucose, which has a hydrodynamic radius of about 0.4 nm (Scherrer and Gerhardt 1971).

While the interaction of nystatin with cell membranes has been extensively studied, less attention has been paid to its ability to pass through the phospholipid bilayer, which remains unclear. It is noteworthy that the transmembrane passage of nystatin is not a requirement for its antimicrobial activity, as it can only cause a lethal imbalance of electrochemical gradients by interacting with the outer cell membrane (Bolard 1986). However, understanding the passage of nystatin through membranes could improve its ability to target intracellular stages of pathogens such as *Leishmania* and *Trypanosoma* amastigotes and certain fungal pathogens with greater efficiency and specificity, and could also provide valuable insight into reducing its side effects in cholesterol-containing membranes, for example in the cells of the renal tubules. Indeed, the transmembrane passage of nystatin in renal tubule cells could disrupt processes in subcellular organelles, such as mitochondria, and induce apoptosis (Zager 2000; Fanos and Cataldi 2001; Varlam et al. 2001). Knowledge that nystatin passes through the membrane could therefore contribute to the development of safer and more effective therapeutic formulations.

**Fig. 1:**
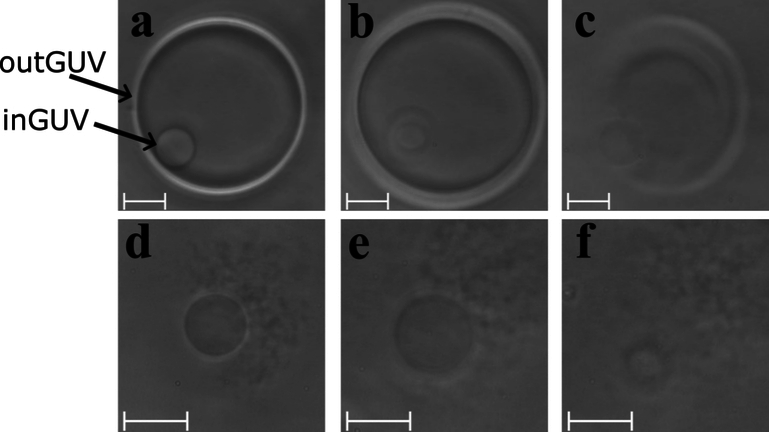
Typical behavior of an MVV (an inGUV within an outGUV) at different points in time. Snapshots were taken **a** 4 s after the transfer of the MVV into the measurement compartment with the bulk solution, **b** just before the rupture of outGUV at 193 s, **c** during the rupture of the outGUV at 194 s, **d** immediately after the rupture of the outGUV at 195 s, **e** just before the rupture of the inGUV at 293 s, and **f** during the rupture of the inGUV at 294 s. The images were taken for the MVV with 45 mol% ergosterol membrane content at 500 $\mu$M nystatin concentration. The scale bar represents 10 $\mu$m

To address this, the behavior of giant multivesicular vesicles (MVVs) after their exposure to a nystatin solution was studied and compared with the behavior of monovesicular giant unilamellar vesicles (GUVs), exposed to the same solution. Each MVV consisted of an outer giant unilamellar vesicle (outGUV) and mostly one smaller giant unilamellar vesicle (inGUV) within the outGUV (Fig. 1). Importantly, the abbreviation GUV refers exclusively to single, unilamellar vesicles that neither encapsulate nor are encapsulated by other vesicles. Furthermore, unless otherwise specified, the term vesicle is used broadly to refer to any membrane-bound lipid structure, regardless of its type. The experiments were performed with nystatin solutions at concentrations of 250 and 500 $\mu$M at which vesicle ruptures frequently occur. During the rupture the vesicle membrane is disintegrated. The differences in rupture times between the inGUVs and the GUVs of comparable size were interpreted as evidence of the ability of nystatin to pass through the membrane of the outGUVs. On this basis, the possible mechanism of passage of nystatin through the phospholipid membrane was proposed. Vesicles with ergosterol-containing POPC membranes can be studied, as they frequently undergo membrane disintegration when exposed to a nystatin solution (Kristanc et al. 2012, 2014). Unfortunately, the same experimental approach cannot be applied to vesicles composed of pure POPC or those containing cholesterol.

## Materials and Methods

### Preparation of Vesicles and Nystatin Solutions

The GUVs and MVVs were prepared from a mixture of palmitoyl-oleoyl-phosphatidyl-choline (POPC) and ergosterol according to the modified method described in (Angelova et al. 1992). The ergosterol (Sigma-Aldrich, USA) was mixed with POPC (Avanti Polar Lipids, USA) in molar ratios of 15:85 and 45:55. The mixtures were dissolved in a 1:1 chloroform-methanol solution, spread on platinum electrodes and dried in a vacuum dryer. The electrodes were placed in an electroformation chamber filled with 2 ml of 0.2 M sucrose solution. An alternating electric field was applied which was gradually reduced from an initial value of 1 V/mm and 10 Hz to a final value of 0.1 V/mm and 1 Hz, resulting in the formation of GUVs filled with the sucrose solution. This method can also lead to the occasional formation of more complex vesicular structures such as MVVs. These structures were relatively rare, with approximately 1 in 40 vesicles being an MVV containing at least one inGUV. Formed vesicles were transferred to the isomolar (0.2 M) glucose solution and stored at a room temperature of 24 ± 1 °C. The samples were used within the second day after their preparation to allow sufficient lateral distribution of ergosterol in the membranes and to avoid degradation of the vesicles.

A 10 mM stock suspension of nystatin was prepared from lyophilized nystatin (Fluka, Sigma, USA) in pure methanol shortly before the experiment. The nystatin solutions with concentrations of 250 and 500 $\mu$M were prepared by diluting the stock suspension with 0.2 M glucose solution and stirred with a vortex mixer.

### Experimental Setup and Procedure

The vesicles were observed with an inverted microscope (Diaphot 200, Nikon, Japan; objective CF Fluor LWD DM 60/0.70) using the phase contrast microscopy technique. Images were acquired continuously using a cooled black-and-white CCD camera (4742-95, Hamamatsu, Japan) controlled by Wasabi image acquisition software. The images of the vesicles were focused in the equatorial plane.

A custom-made measuring cell consisting of two separate compartments was used to manipulate and observe the vesicles. The cell consisted of an acrylic glass plate (dimensions: 8$\times$3 $\hbox {cm}^2$) with two rectangular openings on the longer side—one larger (1$\times$0.8 $\hbox {cm}^2$) and one smaller (1$\times$0.5 $\hbox {cm}^2$). The cover glasses were attached to both the top and bottom of the acrylic plate with silicone glue along the edges of the openings, creating two separate compartments. The first compartment (vesicle compartment with a volume of 250 $\mu$L) was filled with the glucose solution containing the vesicles, and the second compartment (measurement compartment with a volume of 150 $\mu$L) was filled with the glucose-methanol solution containing nystatin at a concentration of 250 or 500 $\mu$M (hereafter bulk solution).

Using a micropipette manipulation system, 1–5 individual spherical vesicles with a diameter between 4 and 70 $\mu$m and without anomalies, such as protrusions, were transferred into the measurement compartment. Micropipettes were prepared using a commercially available borosilicate glass capillaries (WPI, USA). A glass pipette puller (Kopf Instruments, Model 730, USA) and a pipette microforge (Research Instruments Limited, UK) were used to obtain micropipettes with smooth, rounded tips with diameters of 50–100 $\mu$m. The micropipette filled with an isoosmolar 0.2 M glucose solution was mounted on a micromanipulation system using flexible tube. The system consisted of a glass reservoir filled with distilled water, the height of which can be adjusted relative to the micropipette tip with a microscrew to control the pressure to expel or aspirate the solution with vesicles. The volume transferred from the vesicle compartment to the measurement compartment in each experiment was less than 0.02 $\mu$L, i.e., about ten thousand times smaller than the measurement compartment. The vesicles were observed after their transfer into the measurement compartment. The measurements were carried out at room temperature.

The control measurements were carried out in a glucose-methanol solution without nystatin. The methanol concentration was the same as in the experiments with 500 $\mu$M nystatin concentration (1 M). To estimate the contribution of nystatin to the increase in membrane area, additional experiments were performed in a hypotonic glucose solution (0.17 M) without nystatin and without methanol.

### Image Analysis

The images were analyzed using ImageJ software. The rupture times of the vesicles and their radii were measured for each vesicle type, i.e., GUVs, outGUVs and inGUVs. The rupture time of the vesicle was defined as the time between its transfer into the measurement compartment and its rupture (Fig. 1). The time in the bulk solution of the inGUV was calculated by subtracting the rupture time of the corresponding outGUV from the rupture time of the inGUV.

The radius of the vesicle was measured at multiple time points, starting just after transfer into the measurement compartment and ending shortly before rupture. ImageJ was used to select a circular region of interest (ROI) centered on the vesicle in each chosen image. Within the ROI, image intensity profiles were created along multiple radial lines extending outward from the center at evenly spaced intervals over 360°. The radial lines were extended beyond the vesicle membrane, approximately 30% further than the edge of the vesicle. The intensities were recorded at each 0.1° angular step. Since the radial lines frequently crossed the image matrix at subpixel coordinates, the intensity values were interpolated by bilinear interpolation based on the nearest four pixel values. For each radial line, the vesicle membrane was identified by locating the midpoint between the minimum and maximum intensity points along the intensity profile—the region where the slope of the intensity gradient was steepest. Namely glucose and sucrose molecules have slightly different refractive indices (Weast and Astle 1978). Accordingly, the edges of the vesicles appeared darker than the surrounding medium. The vesicle radius was then calculated as the mean radial position of the membrane over all angular steps. For inGUVs before rupture of the corresponding outGUV, the bright halo effect was small because the sucrose solution inside the inGUV is similar to the solution of the surrounding outGUV. In these cases, the position of the membrane was determined directly at the point of minimum intensity along the radial profile. This method was also used for the radius measurements in the control measurements.

Standard statistical procedures including averages, medians, errors of the mean and standard deviations were used to analyze the experimental data. Significance analysis was performed using Student’s *t*-distribution. The two means of the data sets were considered significantly different if the probability value (*p*-value) was less than 0.05.

## Results

### Rupture Times of the Vesicles

**Fig. 2:**
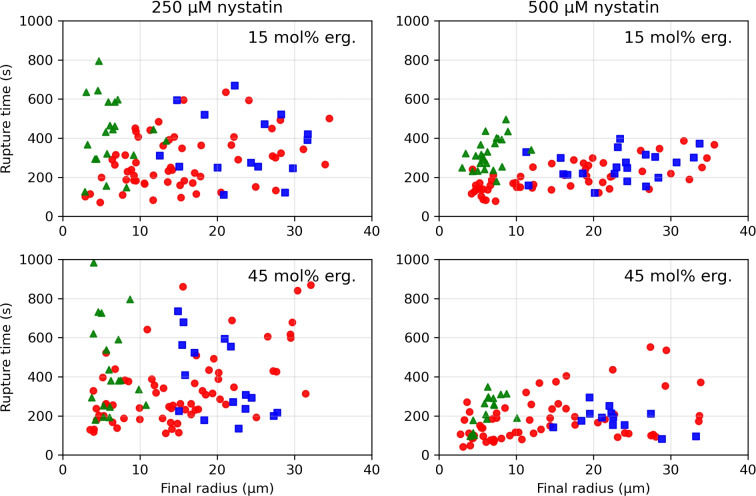
Rupture times, given relative to the time that the vesicles were transferred into the nystatin-containing chamber, as a function of the final radius for GUVs (Filled red circle), outGUVs (Filled blue square) and inGUVs (Filled green triangle). The results are shown for the vesicles with 15 mol% (above) and 45 mol% (below) ergosterol membrane content, and for 250 $\mu$M (left) and 500 $\mu$M (right) nystatin concentration

**Fig. 3:**
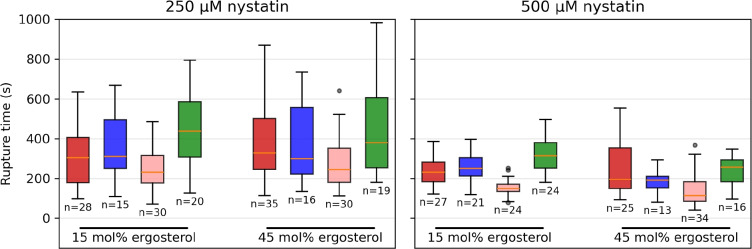
Rupture times of vesicles, given relative to the time that the vesicles were transferred into the nystatin-containing chamber, as a function of their compositions for GUVs with final radii greater than 14 $\mu$m (red), outGUVs (blue), GUVs with final radii smaller than 14 $\mu$m (light red) and inGUVs (green). The radius and the rupture time of the individual vesicle are presented in Fig. 2. In each case, the boxes span over 50 % of the measurements from the mean, the whiskers enclose the range of measurements, and the horizontal lines indicate the medians. The numbers of vesicles (n) of the corresponding type are also given

The rupture times, i.e., the times between the transfer of the vesicles into the measurement compartment and their rupture, as a function of the final radii are shown in Fig. 2, while the rupture times for each vesicle type at each nystatin concentration are summarized in Fig. 3. Firstly, it has to be noted that for none of the observed MVVs did an inGUV rupture before the corresponding outGUV. Secondly, the measurements for both membrane compositions and at both nystatin concentrations show that (i) the GUVs with final radii greater than 14 $\mu$m and the outGUVs of similar size exhibit comparable rupture times, and (ii) the GUVs with final radii bellow 14 $\mu$m exhibit significantly shorter rupture times than the inGUVs with similar final radii. The division of GUVs into small and large GUVs is necessary for a meaningful comparison of GUVs with inGUVs and with outGUVs. The division of the GUVs according to size showed that the GUVs with final radii smaller than 14 $\mu$m have shorter rupture times on average than GUVs with final radii larger than 14 $\mu$m. A radius of 14 $\mu$m was chosen as the boundary, as all inGUVs had final radii smaller than 14 $\mu$m, while almost all outGUVs (95.4%) were larger than 14 $\mu$m. For 15 mol% ergosterol content, the two-tailed *p*-value comparing the rupture times of GUVs larger than 14 $\mu$m to outGUVs is 0.64 at the 250 $\mu$M nystatin concentration and 0.19 at the 500 $\mu$M concentration. For 45 mol% ergosterol, these values are 0.22 at 250 $\mu$M and 0.07 at 500 $\mu$M nystatin concentration, showing no remarkable differences between the GUVs with final radii greater than 14 $\mu$m and outGUVs of similar size. In contrast, the differences in rupture times between the GUVs with final radii below 14 $\mu$m and the inGUVs of similar size are significant. The corresponding two-tailed *p*-values are $6.7 \times 10^{-4}$ at the 250 $\mu$M nystatin solution and $1.0 \times 10^{-10}$ at the 500 $\mu$M solution for 15 mol% ergosterol content. For 45 mol% ergosterol content, the *p*-values are $5.6 \times 10^{-3}$ at 250 $\mu$M nystatin concentration and $9.8 \times 10^{-4}$ at 500 $\mu$M, respectively. Thirdly, it should be noted that the rupture times of all vesicle types are significantly longer at the 250 $\mu$M nystatin concentration than at the 500 $\mu$M concentration with *p*-values of less than 0.04.

### Times in the Bulk Solution

To determine whether the vesicle membrane is permeable to nystatin molecules, the times spent by the inGUVs and the GUVs in the bulk solution until their rupture were compared. They are referred to as the times in the bulk solution. For a GUV, the time in the bulk solution corresponds to its rupture time, whereas for an inGUV, the time in the bulk solution is the time elapsed from the rupture of the outGUV to the rupture of the inGUV.

**Fig. 4:**
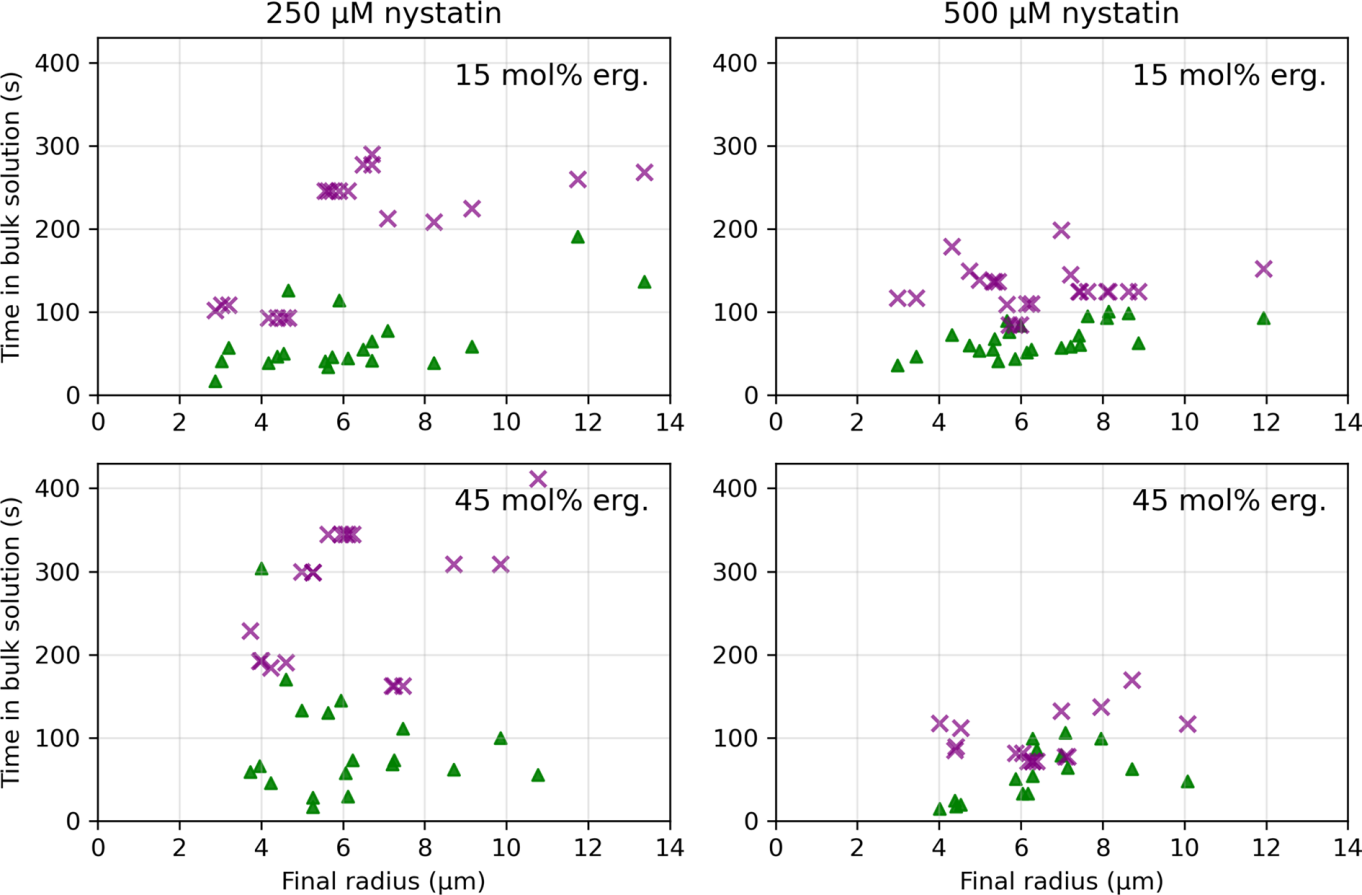
Time in the bulk solution as a function of final radius for inGUVs (Filled green triangle) and GUVs ($\times$) with the same final radii. The results are shown for the vesicles with 15 mol% (above) and 45 mol% (below) ergosterol membrane content, and for 250 $\mu$M (left) and 500 $\mu$M (right) nystatin concentration

**Fig. 5:**
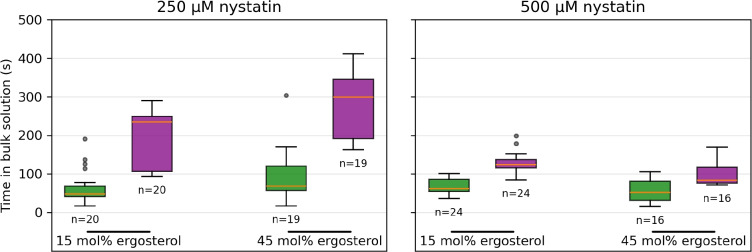
Time in the bulk solution of vesicles as a function of their compositions for the inGUVs (green) and the GUVs with the same final radii (purple). The radius and the time in the bulk solution of the individual vesicle are presented in Fig. 4. In each case, the boxes span over 50 % of the measurements from the mean, the whiskers enclose the range of measurements, and the horizontal lines indicate the medians. The numbers of vesicles (*n*) are also given

The times in the bulk solution for the inGUVs and the GUVs with the same final radii as a function of their final radius are shown in Fig. 4, while their times in the bulk solution at each nystatin concentration are summarized in Fig. 5 for both ergosterol contents. It is to be noted that in cases where the GUV with the same final radius was not available for direct comparison, an average time of a GUV with a slightly larger and a GUV with a slightly smaller final radius was taken.

The data show that the inGUVs spend on average a shorter time in the bulk solution than the GUVs of the same size, regardless of the composition of the membrane. At the 250 and 500 $\mu$M nystatin concentrations, the times in the bulk solution of the inGUVs were 66% ± 25% and 45% ± 25% (*MEAN* ± *SD*) shorter than the average times in the bulk solution of the corresponding GUVs. These differences were statistically significant with two-tailed *p*-values below $10^{-4}$.

It should also be noted that the difference between the times spent in the 250 and 500 $\mu$M nystatin solution is smaller for inGUVs than for corresponding GUVs. The inGUVs spend on average 27% ± 15% (*MEAN* ± *SD*) more time in the bulk solution with 250 $\mu$M nystatin concentration than in the bulk solution with 500 $\mu$M nystatin concentration. In contrast, the corresponding GUVs spend on average 107% ± 32% (*MEAN* ± *SD*) more time in the bulk solution with 250 $\mu$M nystatin concentration than in the bulk solution with 500 $\mu$M.

### Increase of the Vesicle Radius

**Fig. 6:**
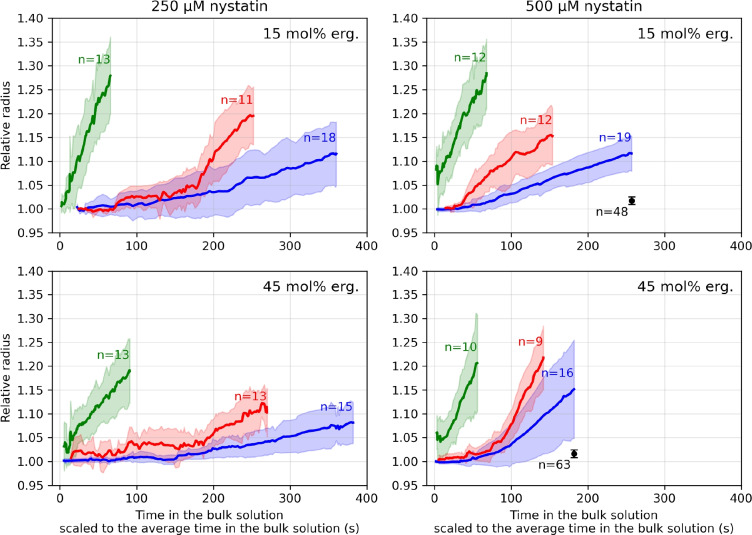
Radii (Mean ± SD) of GUVs with final radii below 14 $\mu$m (red), inGUVs (green) and outGUVs (blue) as a function of time. The radii are given relative to the first measured radius of each vesicle after the transfer into the measurement compartment for each vesicle type. The observed radius increase time is scaled to its average rupture time for GUVs and outGUVs, and to its average time in the bulk solution for inGUVs, which are presented in Fig. 3. The results are shown for the vesicles with 15 mol% (above) and 45 mol% (below) ergosterol membrane content, and for 250 $\mu$M (left) and 500 $\mu$M (right) nystatin concentration. The average relative radius increases measured in the glucose-methanol solution without nystatin are indicated by black dots, with error bars representing the standard deviation. The numbers of vesicle measurements (*n*) per group are indicated in the panels using the corresponding colors

The measurements of the radii of the vesicles, whose rupture times are presented in Fig. 2, in the bulk solutions with the 250 and 500 $\mu$M nystatin concentrations show on average similar critical relative radius increases of 16% ± 7% and 19% ± 6% (*MEAN* ± *SD*) for all vesicle types, respectively. Due to the relatively large standard deviations observed within each group, the differences in the critical relative radius increase among the different vesicle types are not statistically significant. The average time courses of radius increase of inGUVs, outGUVs and GUVs with final radii below 14 $\mu$m containing 15 or 45 mol% ergosterol in 250 or 500 $\mu$M nystatin are shown in Fig. 6. For all vesicle types, the radii are given relative to the first measured radius after the vesicle was transferred to the measurement compartment. In the case of inGUVs, only the courses of the radius increase after the rupture of the corresponding outGUV are shown. For better comparison, the observed time in the bulk solution for each vesicle type with chosen ergosterol content and in chosen solution is scaled to the corresponding average time in the bulk solution, which is shown in Fig. 3.

The incorporation of methanol molecules into the vesicle membrane, as reflected by the increase in vesicle radius, was determined by the experiments in the glucose-methanol solution without nystatin. No ruptures were observed in the vesicles during these experiments. For a meaningful comparison, the radii of the vesicles in the glucose-methanol solution were therefore measured just after the transfer to the measurement compartment and at the mean rupture times of the outGUVs in nystatin solution, which were determined in subsection Rupture times of the vesicle 3.1. The mean rupture time in the presence of nystatin was 308 s for outGUVs with 15 mol% ergosterol content and 282 s for outGUVs with 45 mol% ergosterol content (Fig. 3). The relative radii increases of all vesicle types were similar for 15 and 45 mol% ergosterol membrane content, namely 1.7% ± 0.8% ($MEAN \pm SD$, $n=48$) and 1.6% ± 0.8% ($n=63$) as seen in Fig. 6, respectively. On average, the vesicle radius reached a plateau value (between 2 and 25 $\mu$m) in approximately 20 s after transfer into the glucose-methanol solution, after which no further increase or decrease in the mean radius was observed. No statistically significant differences were found between outGUVs, inGUVs and GUVs in terms of relative radius increase, which is consistent with the high membrane permeability to methanol (Sun et al. 2018).

Furthermore, the measurements performed on the vesicles in the hypotonic glucose solution, i.e., without nystatin and without methanol, show similar critical relative radius increases for 15 and 45 mol% ergosterol membrane content, namely 4.0% ± 1.9% ($MEAN \pm SD$, $n=19$) and 3.8% ± 1.7% ($n=22$), respectively. Here, the radius increases were determined just before the actual rupture of the vesicle.

## Discussion

This study aimed to determine whether and to what extent the channel-forming agent nystatin passes through the ergosterol-containing phospholipid membrane, which serves as a model for the cell membrane. For this purpose, both monovesicular giant unilamellar POPC vesicles (GUVs) and giant multivesicular POPC vesicles (MVVs), which consist of outer (outGUVs) and inner (inGUVs) vesicles, were exposed to nystatin and their rupture times were compared. By comparing the rupture times of GUVs and inGUVs of the same size, the passage of nystatin through the vesicle membrane could be deduced. MVVs can also be used to study the ability of other molecules to passively cross the lipid membrane. In the case of amphipathic polypeptides, their passage was determined by the fluorescence signal that occurs due to the passage of the corresponding dye (Wheaten et al. 2013).

### Explanation of Vesicle Ruptures

A brief explanation of vesicle rupture (e.g., rupture of GUV, outGUV, or inGUV) induced by a channel-forming agent should be given. When the vesicle membrane is exposed to sufficiently high concentrations of the channel-forming agent nystatin, size-selective transmembrane channels are formed (Holz and Finkelstein 1970; Katsu et al. 2008). A selective passage, i.e., a higher flux of smaller glucose molecules compared to larger sucrose molecules, is induced (Kristanc et al. 2014). Since in our experiments the glucose molecules are outside the vesicle and the sucrose molecules are in the inner solution, the number of sugar molecules and, consequently, the sugar concentration inside the vesicle increase. As a result, a net flux of water into the vesicle is induced and the volume of the vesicle increases. When the area of the vesicle reaches its critical value, the vesicle ruptures (Koslov and Markin 1984; Idiart and Levin 2004).

In addition to increasing the permeability for glucose, nystatin also significantly increases the permeability of the membrane for water. Both increases are necessary for the large influx of water (Kristanc et al. 2014). This is because a considerable influx of water is required for vesicle rupture, in which the membrane is completely disintegrated.

### Experimental Evidence of Nystatin Passage

Comparable rupture times of GUVs and outGUVs of the same size show, according to the previous discussion, that the nystatin channels are formed in both types of vesicles over the same time course (Fig. 2). In contrast, the experimental data show that the rupture times of GUVs and inGUVs with the same size are significantly different, indicating a different time course of the nystatin channel formation in their membranes. In addition, their times in the bulk solution, i.e., in the 250 or 500 $\mu$M nystatin concentration, are significantly different. As can be seen in Fig. 4, the time between the exposure of the GUV to the nystatin solution and its rupture is significantly longer than the time between the rupture of the outGUV and the rupture of the inGUV, i.e., the time during which the inGUV is exposed to the bulk solution. The earlier rupture of the inGUVs indicates that, unlike the GUV, the inGUV has already incorporated nystatin into the membrane before it is exposed to the bulk solution, which is the reason why the inGUV ruptures earlier than the GUV. However, this incorporation of nystatin into the membrane of the inGUV is only possible if nystatin molecules pass through the membrane of the outGUV into its interior and bind to the membrane of the inGUV before the outGUV ruptures.

Also, in any of the experiments the rupture of the outGUV did not occur simultaneously or later than the rupture of the corresponding inGUV, even if the radius of the inGUV is much smaller than the radius of the outGUV of the same MVV. This indicates that the nystatin molecules cannot pass the membrane of the outGUV unhindered. In summary, the assumptions that nystatin does not pass through the membrane or that it passes through the membrane instantly, are therefore deficient.

The nystatin passage is also supported by the comparison of the radius increase between inGUVs and GUVs with radii smaller than 14 $\mu$m (Fig. 6). For all tested nystatin concentrations and ergosterol contents, the inGUVs initially show a larger increase in radius immediately after the exposure to the bulk solution than the corresponding GUVs (Fig. 6). The increase in vesicle radius can only be larger if the portion of nystatin molecules in the membrane, causing the greater influx of glucose and water, is greater. The glucose and water molecules can therefore pass through the membrane of the inGUV to a greater extent after rupture of the corresponding outGUV, which indicates that the nystatin is already incorporated into the membrane of the inGUV when the outGUV ruptures.

It should be noted that the average radius shown in Fig. 6 reflects the volume of the vesicle, as the vesicle volume is, to a first approximation, proportional to the cube of its radius. However, the vesicle radius is less relevant for determining the membrane area because an increase in membrane area at constant volume primarily decreases lateral tension and, accordingly, increases membrane fluctuations, which are not directly reflected in an increase in vesicle radius (Seifert 1997).

Additionally, before the outGUV ruptures, there is an influx of glucose through its channels into its interior, which leads to a slightly higher sugar concentration in the solution surrounding the inGUV. This tends to reduce the volume of the inGUV if no nystatin is bound to its membrane. In this case, due to the lower volume of the inGUV at the exposure to the bulk solution, it would take longer for the inGUV to rupture than a GUV of the same size. In contrast, we observe in the experiments that the mean time of the inGUV in the bulk solution is shorter than the mean time of the GUV of the same size in the bulk solution. This conclusion is in favor of the passage of nystatin through the membrane.

The results also show that the radius increases of vesicles exposed to the nystatin solution are considerably large (subsection Increase of the vesicle radius 3.3). The increases could, to some extent, be explained by the excess membrane area, i.e., the reduction of membrane undulations, since our vesicles are not initially tense. Our control measurements showed an approximate 8% increase in membrane area due to reduced membrane undulations and membrane stretching, which corresponds to the contribution of thermal fluctuations and membrane elasticity in lipid membranes (Seifert 1997; Evans et al. 2013). In addition, other effects contributing to increases in membrane area cannot be neglected, including: (i) binding and pore formation by nystatin, (ii) methanol binding, and (iii) changes in the membrane’s stretching constant and lysis tension due to nystatin and methanol binding. Our control measurements showed an approximate 3.3% increase in membrane area due to the effect of methanol, leading to a total increase of about 12% in membrane area without accounting for the effects of nystatin. This leaves approximately 24% of the area increase ascribed to nystatin binding and pore formation, as well as to the changes in the membrane’s stretching constant and lysis tension caused by the intercalation of nystatin and methanol. The density of nystatin pores must be sufficiently high to cause vesicle rupture—approximately one pore per 100 lipid molecules (Kristanc et al. 2014)—which could result in an additional 7% increase in vesicle area. We may also speculate that membrane thinning, a phenomenon similar to that induced by high methanol concentrations, could occur around the nystatin pores, thereby increasing surface area (Simon and McIntosh 1984; Zeng et al. 1993; Kristanc et al. 2012). Nevertheless, it must be emphasized that our experimental determinations of the radius increase, with a standard deviation of approximately 40%, are not accurate and, therefore, are not used as the primary evidence for the passage of nystatin molecules through the membrane. However, these results highlight the substantial impact of nystatin on membrane behavior.

### Proposed Mechanism of Nystatin Passage

According to the well-accepted model of nystatin channel formation, nystatin aggregates interact with the phospholipid membrane and dissolve, allowing monomers or small oligomers to bind horizontally to the outer layer of the phospholipid bilayer (Zemel et al. 2005; Cohen 2010; Szomek et al. 2021). At a sufficient membrane density, these monomers or small oligomers undergo oligomerization and reorientation perpendicular to the membrane surface (Recamier et al. 2010), which is facilitated by the amphipathic nature of nystatin. Once embedded, they continue to oligomerize, forming transmembrane channels (Kleinberg and Finkelstein 1984; Zemel et al. 2005). The number of these channels and the number of nystatin monomers composing the individual channel can vary over time.

**Fig. 7:**
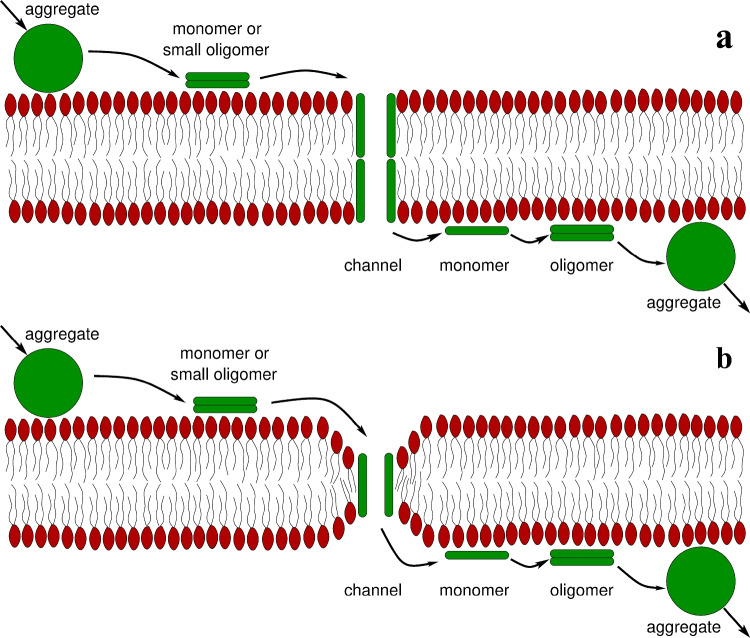
Schematic representation of the mechanism that enables the passage of nystatin molecules through the POPC membrane. A passive net flux of nystatin molecules across the membrane from the side with a higher nystatin concentration to the side with a lower concentration is denoted by the arrows. This flux is characterized by the binding of aggregates, the dissociation of monomers or small oligomers from aggregates, the formation of channels, the dissociation of single molecules from the channels, the formation of oligomers and aggregates, and the unbinding of molecular aggregates. The representation corresponds to two possible ways in which nystatin spans the lipid bilayer: by a tandem insertion into both monolayers (**a**) or by a conformational thinning of the membrane (**b**)

Based on this model, the following mechanism is proposed for the passage of nystatin through the phospholipid bilayer: some monomers can dissociate from the individual channels and can move into the solution on both sides of the membrane (Fig. 7). Consequently, a passive net flux of nystatin molecules is induced across the membrane from the side with a higher nystatin concentration to the side with a lower concentration. The proposed mechanism predicts that the passage of nystatin molecules through the membrane only occurs when the nystatin channels are formed in the membrane.

The proposed mechanism is supported by the experiments performed at high nystatin concentrations (above 250 $\mu$M), where the formation of nystatin channels leads to the ruptures of the vesicles. However, previous studies have shown that nystatin channels are also formed at much lower nystatin concentrations, below 25 $\mu$M (Recamier et al. 2010). We postulate that the mechanism for the passage of nystatin molecules through the membrane may be operational at lower nystatin concentrations, where channel formation still occurs, although vesicle ruptures are absent or occur far less frequently. These lower nystatin concentrations are often physiologically relevant. Nystatin concentrations in internal organs such as the kidneys and liver typically remain much lower—in the range of 1–10 $\mu$M (Semis et al. 2012). In contrast, after intravenous application, nystatin concentrations can reach up to 35 $\mu$M in the bloodstream, and after topical application, levels may reach up to 200 $\mu$M in the upper layers of the skin or mucosal surfaces (Groll et al. 2003; Semis et al. 2012). It is to be noted that the passage of nystatin molecules by this mechanism is impossible at nystatin concentrations where no transmembrane channels are formed due to an insufficient areal density of nystatin molecules on the membrane surface (Recamier et al. 2010). Therefore, our conclusions do not contradict the literature where no nystatin permeability in lipid membranes was observed.

### Role of Ergosterol Content and Nystatin Concentration

The role of ergosterol lies in enhancing the solubility of nystatin in the membrane (Szomek et al. 2021). In particular, membrane structure, which varies with sterol content, is involved in the solubility of nystatin aggregates in the membrane (Recamier et al. 2010), but the nystatin channels do not require sterol for their formation. This effect could be due to complex, non-linear dynamics, as suggested in previous studies (González-Damián and Ortega-Blake 2010). Therefore, the role of sterol-dependent membrane structure is crucial for polyene activity. However, our experimental results showed nonsignificant differences in average rupture times and average radius increase between vesicles with 15 and 45 mol% ergosterol membrane contents at the same nystatin concentration, with variations falling within the standard deviation of the mean. It must be taken into account that the scatter of the measured rupture times is large. In addition to a data scatter due to biological variability, the scatter due to the random occurrence of ruptures appears (Chabanon et al. 2017).

Further experimental evidence supporting the passage of nystatin through the phospholipid membrane is the significantly smaller differences in the times in the bulk solution between 250 and 500 $\mu$M concentrations for the inGUVs compared to the GUVs (Figs. 4 and 5). Namely, because nystatin passes through the phospholipid membrane of the outGUVs, the key distinction between GUVs and inGUVs is that inGUVs already have nystatin incorporated into their membranes at the beginning of their exposure to the bulk solution, whereas this is not the case for GUVs. The relatively small difference in the times in the bulk solution between 250 and 500 $\mu$M concentration for the inGUVs thus suggests that the process of nystatin incorporation into the membrane of the inGUVs is largely complete at both 250 and 500 $\mu$M nystatin concentration before the outGUV ruptures. It could be speculated that after the rupture of the outGUV, i.e., after the exposure of the inGUV to the bulk solution, the rupture times of inGUVs are mainly influenced by glucose and water influxes through the already formed nystatin channels. In contrast, the GUVs that start without bound nystatin when exposed to the bulk solution show a stronger effect of nystatin concentration on channel formation, resulting in significantly shorter rupture times at 500 $\mu$M than at 250 $\mu$M concentration.

Accordingly, a lower limit for the membrane permeability of nystatin can be estimated on the basis of the rupture times (Fig. 3), assuming that glucose can pass through the membrane unhindered (Supplementary Information). This limit is in the order of magnitude 0.1 $\mu$m/s, considering that the permeability of the membrane for nystatin (*P*) is defined by the equation, $j = P\Delta c$, where *j* is the particle flux density through a membrane and $\Delta c$ is the concentration difference between the two sides of the membrane (Fick’s law).

## Conclusion

This study aimed to elucidate whether nystatin passes through the phospholipid membranes. Understanding this passage is important in order to target the intracellular stages of pathogens more efficiently and to reduce the toxicity of nystatin.

The passage of nystatin was assessed by measuring the rupture times of GUVs and multivesicular vesicles consisting of one outer (outGUV) and one or two inner GUVs (inGUVs). The longer rupture times of the GUVs compared to the rupture times of the inGUVs after the exposure to the bulk nystatin solution demonstrate that the nystatin molecules pass through the membranes of the outGUVs and are already incorporated into the membrane of the inGUVs, while the inGUVs are still inside the outGUVs. Significantly smaller differences in the times in the bulk solution between 250 and 500 $\mu$M concentrations for the inGUVs compared to the GUVs provide additional support for the passage of nystatin through the phospholipid membrane.

Furthermore, a mechanism for the passage of nystatin molecules through the lipid membrane of the vesicle is proposed. The mechanism is based on the formation of nystatin channels that allow some monomers to dissociate from the individual channels and pass into the solution surrounding the membrane. This induces a passive net flux of nystatin molecules through the membrane from the side with a higher concentration of nystatin to the side with a lower concentration.

## Supplementary Information

Below is the link to the electronic supplementary material.

Supplementary file 1 (pdf 117 KB)

## Data Availability

No datasets were generated or analyzed during the current study.
