# Supplementary material for: Passage of the Channel-Forming Agent Nystatin Through Ergosterol-Containing Lipid Membranes
Source: J Membr Biol. 2025 Jul 7;258(5):415–27. doi: 10.1007/s00232-025-00354-3 (PMC12464136; doi:10.1007/s00232-025-00354-3)
Supplement: Supplementary file 1 — (pdf 117 KB) [file 232_2025_354_MOESM1_ESM.pdf]

Passage of the channel-forming agent nystatin through ergosterol-containing lipid membranes

The Journal of Membrane Biology

M. Tinev, L. Kristanc, G. Gomiščεk, B. Božič*

*Institute of Biophysics, Faculty of Medicine, University of Ljubljana, Vrazov trg 2, Ljubljana, 1000, Slovenia,
E-mail: bojan.bozic@mf.uni-lj.si

Estimation of the membrane permeability

Membrane passage can be described quantitatively to a certain extent, since nystatin passage is a complex phenomenon. Membrane permeability can be evaluated under certain assumptions. Firstly, the relative increase in vesicle volume (ε), which leads to membrane tension and consequently to rupture, is proportional to the integral of the nystatin concentration in the vesicle surroundings (c) over time for a given difference in concentration of the sugar molecules

$$\varepsilon = \alpha \int c \, \mathrm{d}t \,, \tag{SI1}$$

where α is the proportionality constant that depends on the composition of the membrane. Secondly, according to Fick's law, we assume that the flux of nystatin molecules into the outGUV through its membrane is proportional to the difference between the nystatin concentrations in the bulk solution (c_0) and the solution in the outGUV. Consequently, the concentration in the outGUV (c_{outGUV}), which depends on the permeability of the outGUV membrane (P), approaches the value of the bulk solution exponentially

$$c_{\mathrm{outGUV}} = c_0 (1 - \mathrm{e}^{-PAt/V}) \,, \tag{SI2}$$

where V is the volume of the outGUV, A is its area and t is the time. Therefore, the expressions for the relative volume increase of the inGUV and the GUV of similar size and composition can be written in the forms

$$\varepsilon_{\mathrm{inGUV}} = \alpha \int_0^{t_{\mathrm{outGUV}}} c_{\mathrm{outGUV}} \, \mathrm{d}t + \alpha \int_{t_{\mathrm{outGUV}}}^{t_{\mathrm{inGUV}}} c_0 \, \mathrm{d}t \tag{SI3}$$

and

$$\varepsilon_{\mathrm{GUV}} = \alpha \int_0^{t_{\mathrm{GUV}}} c_0 \, \mathrm{d}t \,, \tag{SI4}$$

where t_{inGUV} is the rupture time of the inGUV, t_{outGUV} is the rupture time of the outGUV and t_{GUV} is the rupture time of the GUV of similar size to inGUV. If we consider that

inGUVs and GUVs of the same size achieve the same relative volume increase on average ($\varepsilon_{\mathrm{GUV}} = \varepsilon_{\mathrm{inGUV}}$), we obtain

$$t_{\mathrm{inGUV}} - \frac{V}{PA}(1 - \mathrm{e}^{-PAt_{\mathrm{outGUV}}/V}) = t_{\mathrm{GUV}} \,. \tag{SI5}$$

If we also consider that an average outGUV is spherical and has a size of about 20 μm, we obtain values between 0.025 and 0.06 μm/s for the permeability of the membrane using the average rupture times (Figure 3, main text). This estimate of the membrane permeability is underestimated because the glucose concentration in the outGUV is lower than the glucose concentration in the bulk solution. The value for P is about a thousand times lower than the membrane permeability for water and about a hundred thousand times higher than the membrane permeability for glucose [1, 2].

References

[1] Huster, D., Jin, A. J. , Arnold, K., Gawrisch, K.: Water permeability of polyunsaturated lipid membranes measured by ^{17}O NMR. Biophysical Journal **73**, 855–864, (1997)

[2] Wood, R.E., Wirth Jr, F.P., Morgan, H.E., Glucose permeability of lipid bilayer membranes. Biochimica et Biophysica Acta (BBA)-Biomembranes **163**, 171–178 (1968)
